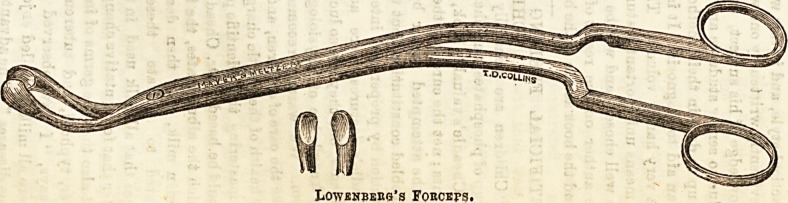# The Treatment of Post Nasal Adenoids

**Published:** 1892-11-26

**Authors:** 


### CENTRAL LONDON THROAT AND EAR HOSPITAL.

### The Treatment of Post Nasal Adenoids.

In dealing with the treatment of inflammatory diseases of the middle ear, we must remember that, speaking generally, there are four varieties of inflammation which occur in this situation—namely, acute and chronic non-suppurative, and acute and chronic suppurative catarrh.

Before, however, saying anything as to the treatment of each of these classes of cases, it will be most convenient to speak of the treatment of the conditions of the pharynx, and naso-pharynx, on which the occurrence of these inflammations so frequently depend—namely, adenoid vegetations and enlarged tonsils.

At the Central London Throat Hospital the removal of adenoid vegetations and tonsillotomy are usually done in the out-patient room, unless the patient lives at too great a distance, in which case he may be taken into the hospital for a time. The patients are, it need hardly be said, in the majority of cases children. The method adopted is as follows: The mouth is kept open by means of a gag, which can be easily moved to either one side or the other (*see diagram*), and the nitrous oxide gas is administered. So soon as the patient is well under, the tonsils are removed by means of a Mackenzie's guillotine, or, if they should be of very large size, by means of a cold wire écraseur, as this much reduces the size of the bleeding surface. If the écraseur be used the anæsthesia does not last long enough, so it is the more usual to remove the tonsils first, without an anæsthetic, and then give gas afterwards for the adenoids.

Wyatt Wingrave's Gag.

The adenoids are dealt with in two or three different ways, according to the operator. One way is to use curettes of different shapes, passed up behind the soft

Post Nasal Curettes.

Lowenberg's Forceps.

palate, or else the growths may be first scraped together with the finger-nail, and then the mass removed by Lowenberg's forceps. Whichever method be used, the last part of the operation always consists in a free scraping with the finger-nail.

The advantage claimed for the use of gas, over chloroform or ether, is that, as the patient comes round immediately, he is able, by coughing, to materially assist in preventing blood and débris from passing into the trachea. The after-treatment consists in preventing the patient from being exposed to cold, by keeping him indoors for about a week. Ice may be sucked at first, as it relieves pain and prevents bleeding, and, of course, the food must, immediately after the operation, consist entirely of slops, and for two or three days should contain nothing rough, such as a crust of bread, to irritate the raw surface. Troublesome bleeding from the tonsil wound is, we believe, practically unknown at the Central London.

*(To be continued.)*

## Figures and Tables

**Figure f1:**
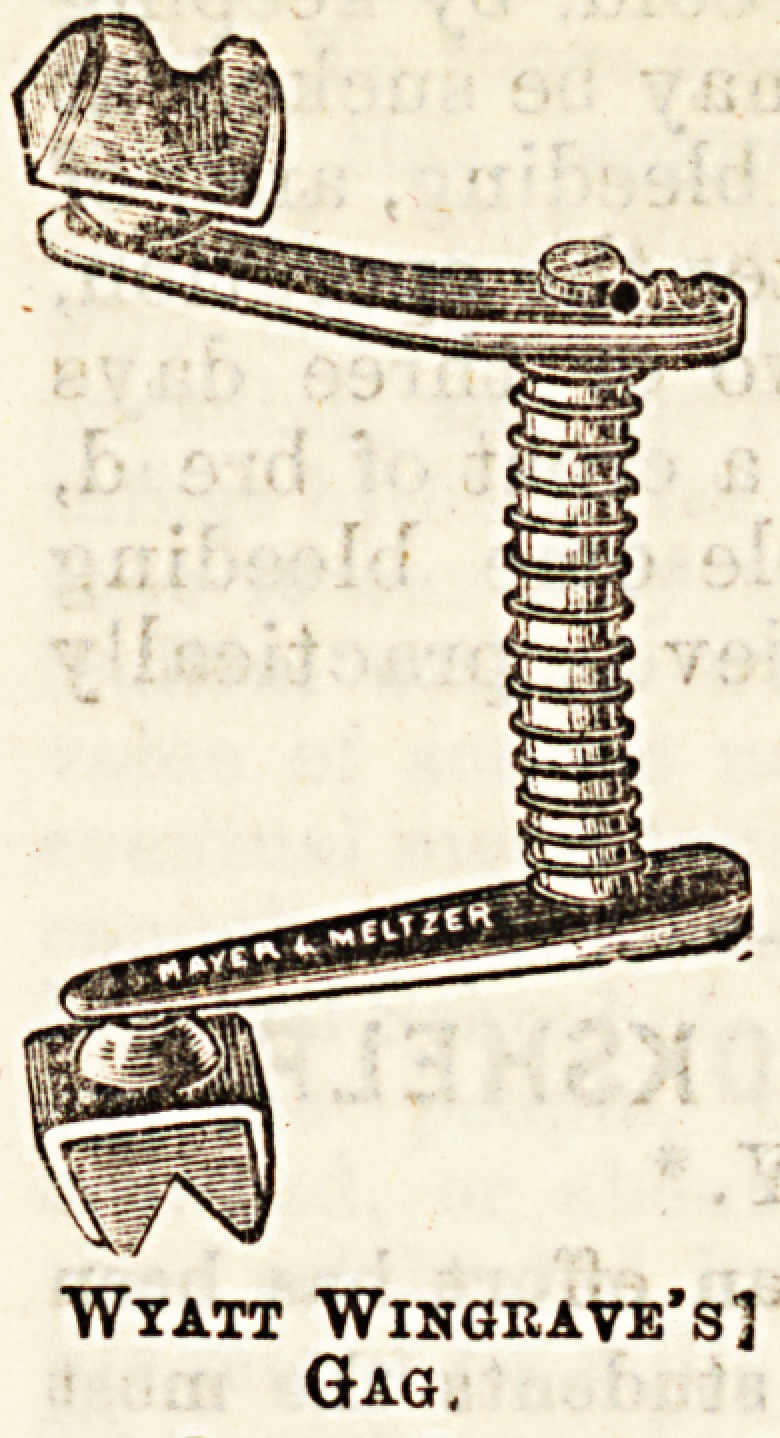


**Figure f2:**
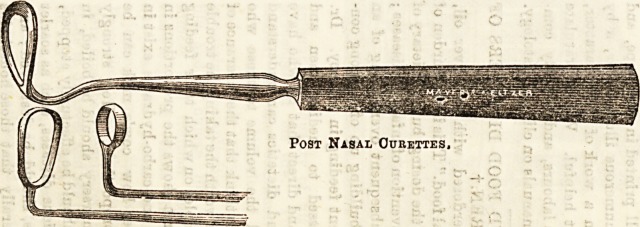


**Figure f3:**